# When a “Replication” Is Not a Replication. Commentary: Sequential Congruency Effects in Monolingual and Bilingual Adults

**DOI:** 10.3389/fpsyg.2019.00797

**Published:** 2019-04-12

**Authors:** John G. Grundy, Ellen Bialystok

**Affiliations:** ^1^Department of Psychology, Iowa State University, Ames, IA, United States; ^2^Department of Psychology, York University, Toronto, ON, Canada

**Keywords:** bilingualism, executive function, bilingual advantage, cognitive control, eriksen flanker task, sequential congruency effect

The importance of replication in psychology, and science more broadly, cannot be overstated given the current state of the literature. The Open Science Collaboration (2015) attempted to replicate 100 studies in three top psychology journals and found that only 36% of effects were replicated. Given this alarmingly low number of successful replications, it is imperative to ensure that when attempting a replication that task parameters and environmental conditions be as similar as possible to the original study in order to determine the reliability and strength of a given effect. Recently, Goldsmith and Morton (2018) claimed a “failed replication” of a study by Grundy et al. (2017) showing that monolinguals were more affected by the influence of the previous trial on current trial performance than bilinguals during a flanker task. The result across three experiments in the original study was that monolinguals had larger sequential congruency effects (SCE; Gratton et al., 1992) than bilinguals in both the behavioral and electrophysiological outcomes. However, the Goldsmith and Morton study that did not replicate this pattern differed from the original experiments in several ways.

The first issue is that Goldsmith and Morton (2018) used a long (1,000 ms) rather than a short (<500 ms) response-to-stimulus interval (RSI). In the original study, Grundy et al. reported that monolinguals had larger behavioral SCEs than bilinguals only at short RSIs and not reliably at longer ones. This point was clearly made in the Abstract: “This finding was strongest at the shortest response-to-stimulus interval (RSI) … Study 3 showed that at long RSIs, where behavioral SCE differences between groups disappear because of sufficient time to recover from the previous trial, event-related potentials demonstrated a continued influence of previous trial congruency for monolinguals but not bilinguals” (Grundy et al., 2017, p.42). The long RSIs that Grundy et al. (2017) are referring to in which there are unreliable behavioral differences are 1,000 ms or more, arguing that the most reliable effects appear when the RSI is 500 ms or less. A group difference at the 1,000 ms RSI only appeared in Study 1 for one of the two tasks and did not appear at all in Study 3 when the RSIs were varied between 1,000 and 1,500 ms. Although it is possible that the lack of group effect in Study 3 was the result of including RSIs >1,000 ms rather than the 1,000 ms RSI itself, it nonetheless highlights the unreliability of longer RSIs. Thus, it appears that Goldsmith and Morton replicated the Grundy et al. findings whereby group effects are least reliable at a 1,000 ms RSI. However, because Goldsmith and Morton only used a single fixed RSI of 1,000 ms, they cannot say anything about what happens at shorter RSIs where the previous studies showed the effects to be most reliable.

Second, Grundy et al. Study 3 (2017) showed that when the RSI was long and behavioral differences were absent between groups, electroencephalography (EEG) revealed the same pattern as the behavioral evidence for short RSIs. Specifically, event-related potentials (ERPs) at the N2 and P3 indicated that monolinguals were more influenced by the previous trial than bilinguals. Goldsmith and Morton did not collect EEG data so have incomplete evidence about performance at their chosen RSI of 1,000 ms.

These two points are crucial in understanding the explanation offered by Grundy et al. (2017). They argued that because of their experience disengaging attention from languages, contexts, and interlocutors, bilinguals should also be faster at disengaging attention from non-linguistic tasks like the flanker. This faster disengagement would be evident in the form of a smaller SCE for bilinguals than monolinguals but that behavioral group differences were less likely to occur at long RSIs when enough time had elapsed for *all* participants to have disengaged attention from the previous stimulus. These are simple tasks, and 1 s is a long time; in retrospect we should not have expected group differences after 1 s since the relevant processing occurs in the initial stages of stimulus onset. Our finding is that when the RSI is short, bilinguals have resolved the conflicting stimulus information but monolinguals have not, making these trials the critical place where behavioral group differences appear. The more detailed evidence from EEG shows that these disengagement processes occur more rapidly by bilinguals than monolinguals at long RSIs as well.

The final issue is that the questionnaire used to assess bilingualism in Goldsmith and Morton's study was substantially less detailed than the one used by Grundy et al., and information that is required to make a reliable classification of language group was missing. Goldsmith and Morton used a 7-item questionnaire that they claimed was similar to the questionnaire used by Grundy et al. but the instrument used in the original study, the Language and Social Background Questionnaire (LSBQ; Anderson et al., 2018), is an extensive questionnaire containing dozens of items, including information about proficiency as well as environmental context. Goldsmith and Morton provide no information on second-language proficiency, which is a critical second-language component. Several studies have shown that varying levels of second language proficiency affect behavioral outcomes on executive function tasks (e.g., Mishra et al., 2012; Iluz-Cohen and Armon-Lotem, 2013; Singh and Mishra, 2013), so this information needs to be provided.

In sum, Goldsmith and Morton claimed that they failed to replicate the three studies reported by Grundy et al. (2017) in which bilinguals showed smaller SCEs than monolinguals. Claims about replication must follow strict guidelines to ensure that task parameters and conditions are similar across studies so that the results of the study can be compared. However, Goldsmith and Morton used long rather than short RSIs that Grundy et al. showed in their paper were least reliable in producing behavioral differences; they did not include EEG evidence that Grundy et al. used to demonstrate these effects in the absence of behavioral differences; and they had incomplete information about bilingual experience, leading to the possibility that the language group classifications were not sufficiently well-defined. Replication in psychology is an important issue, but in order to determine the strength and reliability of effects, we need to first ensure that we are replicating the conditions of the original study. Without doing so, we risk committing Type II errors and making unjustified conclusions. Ironically, Goldsmith and Morton do essentially provide a replication of one of the points originally reported by Grundy et al. namely, that at long RSI intervals, language group differences in disengagement of attention are less reliable.

## Author Contributions

Both authors listed have made a substantial, direct and intellectual contribution to the work, and approved it for publication.

### Conflict of Interest Statement

The authors declare that the research was conducted in the absence of any commercial or financial relationships that could be construed as a potential conflict of interest.
